# Maternal uniparental disomy of chromosome 15 with concurrent paternal non-chromosome 15 marker chromosome: a rare presentation of prader-willi syndrome

**DOI:** 10.1186/s13039-025-00726-3

**Published:** 2025-08-29

**Authors:** Yang Nannan, Yang Yang, Wang Yan, Wang Hao

**Affiliations:** 1 Prenatal Diagnosis Center, Hangzhou Women’s Hospital (Hangzhou Maternity and Child Health Care Hospital), Hangzhou, Zhejiang China; 2 Assisted Reproduction Unit, Department of Obstetrics and Gynecology, Sir Run Run Shaw Hospital, School of Medicine, Zhejiang University, Hangzhou, 310016 Zhejiang China; 3 Zhejiang Provincial Clinical Research Center for Reproductive Health and Disease, Hangzhou, 310016 Zhejiang China; 4 Zhejiang Key Laboratory of Precise Protection and Promotion of Fertility, Hangzhou, 310016 Zhejiang China

**Keywords:** Chromosomal microarray analysis, Prader-Willi syndrome, Loss of heterozygosity, Uniparental isodisomy, Prenatal diagnosis

## Abstract

**Background:**

Prader-Willi Syndrome (PWS) is a complicated genetic disorder demonstrating a variety of clinical phenotypes. Using molecular cytogenetics approaches to detect the deletions of the paternal 15q11-q13 region and maternal uniparental disomy of chromosome 15 plays an important role in the prenatal diagnosis of PWS.

**Case presentation:**

A pregnant woman with advanced maternal age underwent amniocentesis. The amniotic fluid was subjected to karyotyping and chromosomal microarray analysis. A marker without autosomal material and loss of heterozygosity (LOH) of 15q14-q23 were found in the fetus. The LOH was consistent with maternal uniparental isodisomy (UPD) and the marker was inherited from the father. Methylation-specific multiplex ligation-dependent probe amplification (MS-MLPA) found increased methylation in the fetal 15q11.2-q13 region and fluorescence in situ hybridization confirmed the marker was not originated from chromosome 15.

**Conclusion:**

We presented a rare PWS case showing maternal UPD of chromosome 15 with concurrent paternal marker chromosome in the prenatal setting.

## Background

Prader-Willi Syndrome (PWS) is a complex genetic disorder characterized by a spectrum of physical, mental, and behavioral features [1]. PWS initially manifests in infancy with marked hypotonia and feeding difficulties, and it typically progresses to hyperphagia followed by obesity in later childhood or early adulthood [2, 3]. Additional characteristic features include short stature, hypogonadism, cognitive impairments, and distinctive behavioral phenotypes characterized by temper tantrums, stubbornness, and compulsive behaviors [4]. In Chinese children with PWS, infant malnutrition is a significant manifestation [5, 6].

The fundamental cause of PWS is the lack of active paternal alleles in the 15q11-q13 region, which is associated with genomic imprinting and exhibits gene expression determined by the parental origin. The genetic cause of PWS is primarily characterized by deletions at the paternal 15q11-q13 locus (accounting for 65%-75% of cases), maternal uniparental disomy (UPD) of chromosome 15 (in 20%-30% of cases), and less commonly, imprinting defects (comprising 1%-3%) [7–9]. In some instances, translocations or duplications can also disrupt the normal gene expression within this crucial region [10].

Chromosomal microarray analysis (CMA) serves as the foundational diagnostic tool for PWS, enabling the identification of deletions within the 15q11-q13 region [10]. Methylation-specific multiplex ligation-dependent probe amplification (MS-MLPA) could detect aberrant methylation patterns that indicate UPD or imprinting defects [11]. Fluorescence in situ hybridization (FISH) [12] and next-generation sequencing (NGS) [13, 14] offer detailed analysis of chromosomal abnormalities and identify causative mutations. This report describes a rare PWS case originating from maternal uniparental disomy of chromosome 15, accompanied by an additional marker chromosome that does not originate from chromosome 15.

## Case presentation

**Fig. 1:**
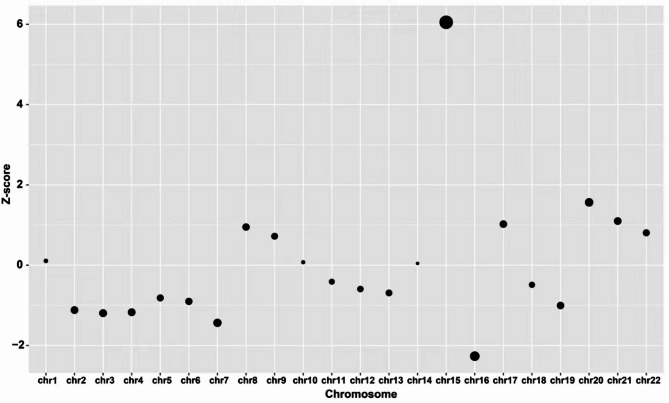
Autosomal Z-score chart of the true-positive sample with mosaic trisomy 15. The Z-score for chromosome 15 is 6.05. The X-axis represents chromosomes 1–22, while the Y-axis represents the corresponding Z-score values. The black dot size is proportional to the absolute value of Z-score

A 41-year-old pregnant woman sought consultation due to advanced maternal age and the detection of an echogenic intracardiac focus in the left ventricle of the fetus’s heart. At 17 weeks’ gestation, she underwent comprehensive non-invasive prenatal testing (NIPT-U+), screening for common autosomal aneuploidies (T21, T18, T13), numerical anomalies of sex chromosomes, all 21 autosomal monosomies, and more than 100 syndromes associated with submicroscopic deletions or duplications exceeding 3 Mb. The NIPT results suggested an increased risk for fetal trisomy 15 (Fig. 1). The couple had previously given birth to a healthy child and experienced one unexplained miscarriage.

**Fig. 2:**
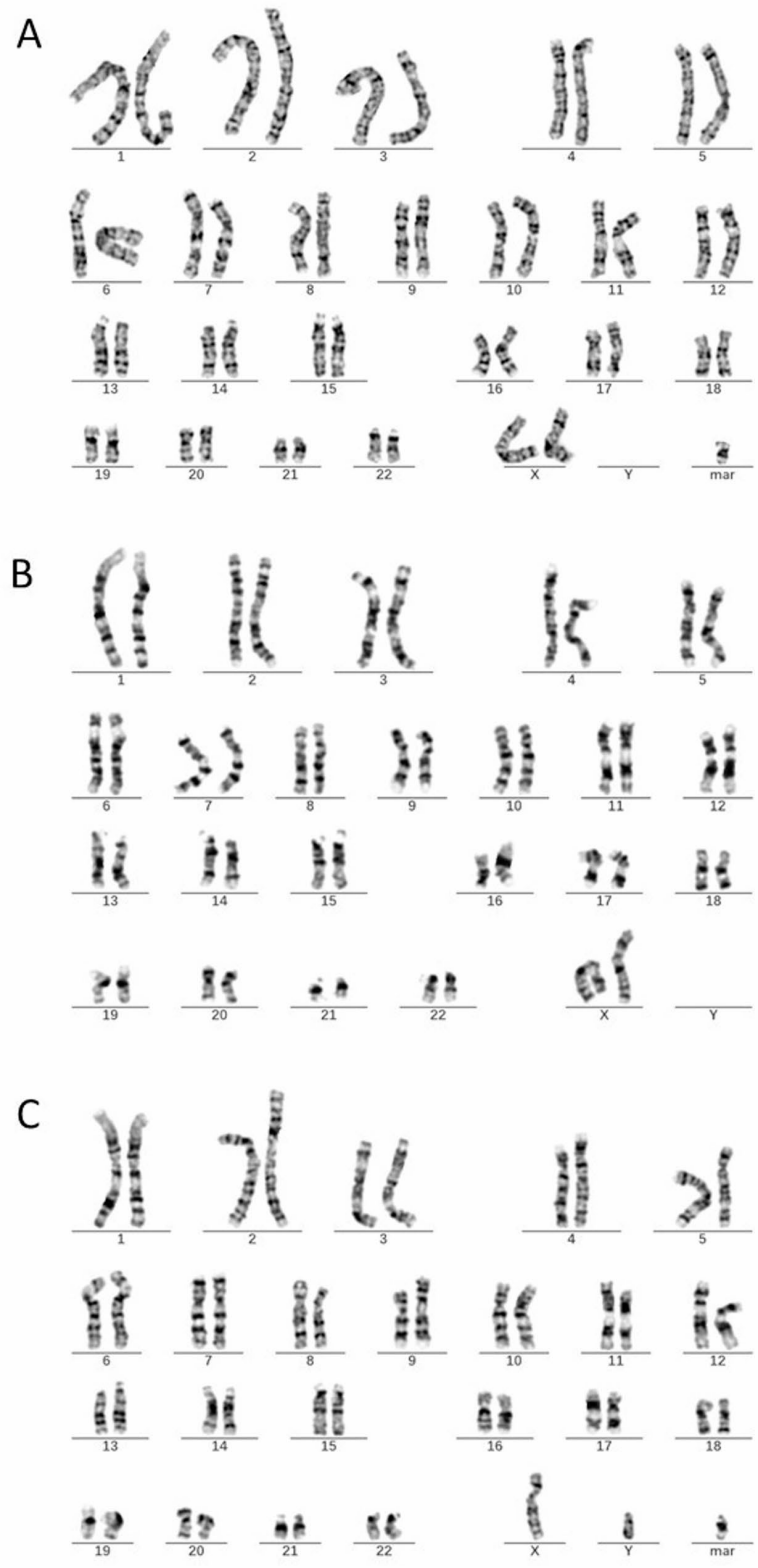
**A**: The fetal karyotype shows an additional marker chromosome. **B**: The maternal karyotype shows no abnormalities. **C**: The paternal karyotype shows the same marker chromosome as observed in the fetus

**Fig. 3:**
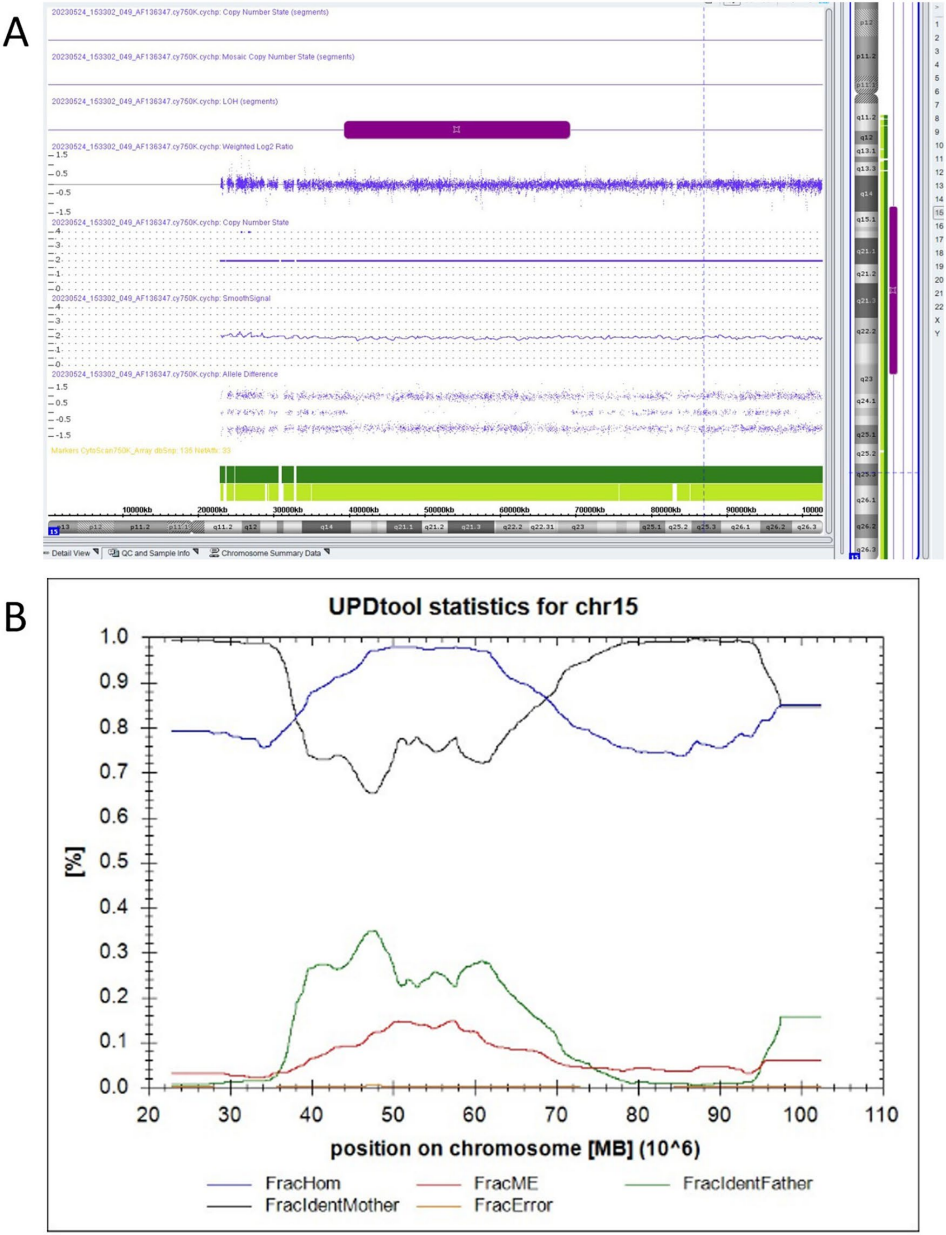
**A**: CMA of the fetal amniotic fluid sample shows Loss of Heterozygosity (LOH) in the segment of chromosome 15. **B**: The fraction of Mendelian errors (MEs) in a 1k-window (FracME = red line = fraction of MEs in a 1k-window) is elevated, indicating UPD. There are two regions (22 Mb-39 Mb and 69 Mb-98 Mb) showing a high fraction of genotypes identical to the mother (FracIdentMother = black line = fraction of genotypes within a 1k-window where both alleles are identical to the mothers’ alleles), indicating maternal hUPD. Between the two hUPD region, maternal iUPD is identified because of the high fraction of homozygous genotypes (FracHom = blue line = fraction of genotypes in 1k-window that is homozygous). FracIdentFather = green line = fraction of genotypes within a 1k-window where both alleles are identical to the fathers’ alleles. FracError = yellow line = fraction of errors (= ME that cannot be explained by UPD) in 1k-window

**Fig. 4:**
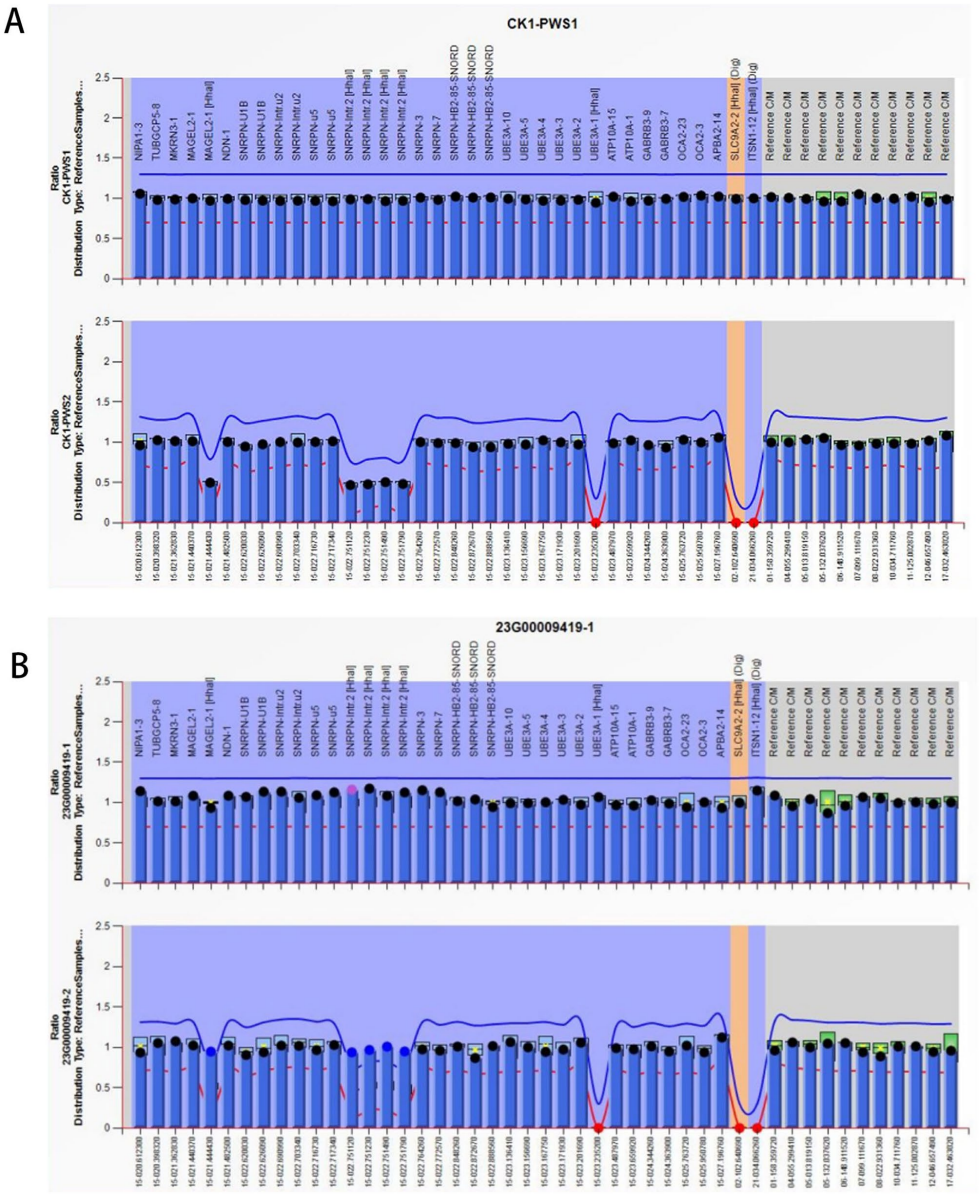
Methylation-Specific Multiplex Ligation-dependent Probe Amplification (MS-MLPA) analysis conducted on the fetal amniotic fluid reveals aberrant methylation patterns. **A**: Reference. **B**: Sample; the upper part of the picture shows the CNV ratio of sample to reference, and the lower part shows the methylation ratio of sample to reference. SNRPN-DMR is hypermethylated compared to the reference (A), indicating maternal UPD

Subsequent amniocentesis was conducted at 20-week gestation. The amniotic fluid samples were subjected to karyotyping and CMA (Affymetrix CytoScan 750 K Array). Karyotyping revealed a female fetus carrying a marker chromosome, which was identified as an inverted duplication marker inherited from the father (Fig. 2). The CMA demonstrated a 29.9 Mb loss of heterozygosity (LOH) in the 15q14-q23 region (Fig. 3A), including genes associated with autosomal recessive conditions such as *EIF2AK4*, *BUB1B*, and *IVD*. Parental CMA analysis of peripheral blood showed no copy number variations; however, the fetal chromosome 15q14-q23 LOH was consistent with maternal uniparental isodisomy (Fig. 3B). MS-MLPA further confirmed increased methylation in the fetal 15q11.2-q13 region (Fig. 4).

**Fig. 5:**
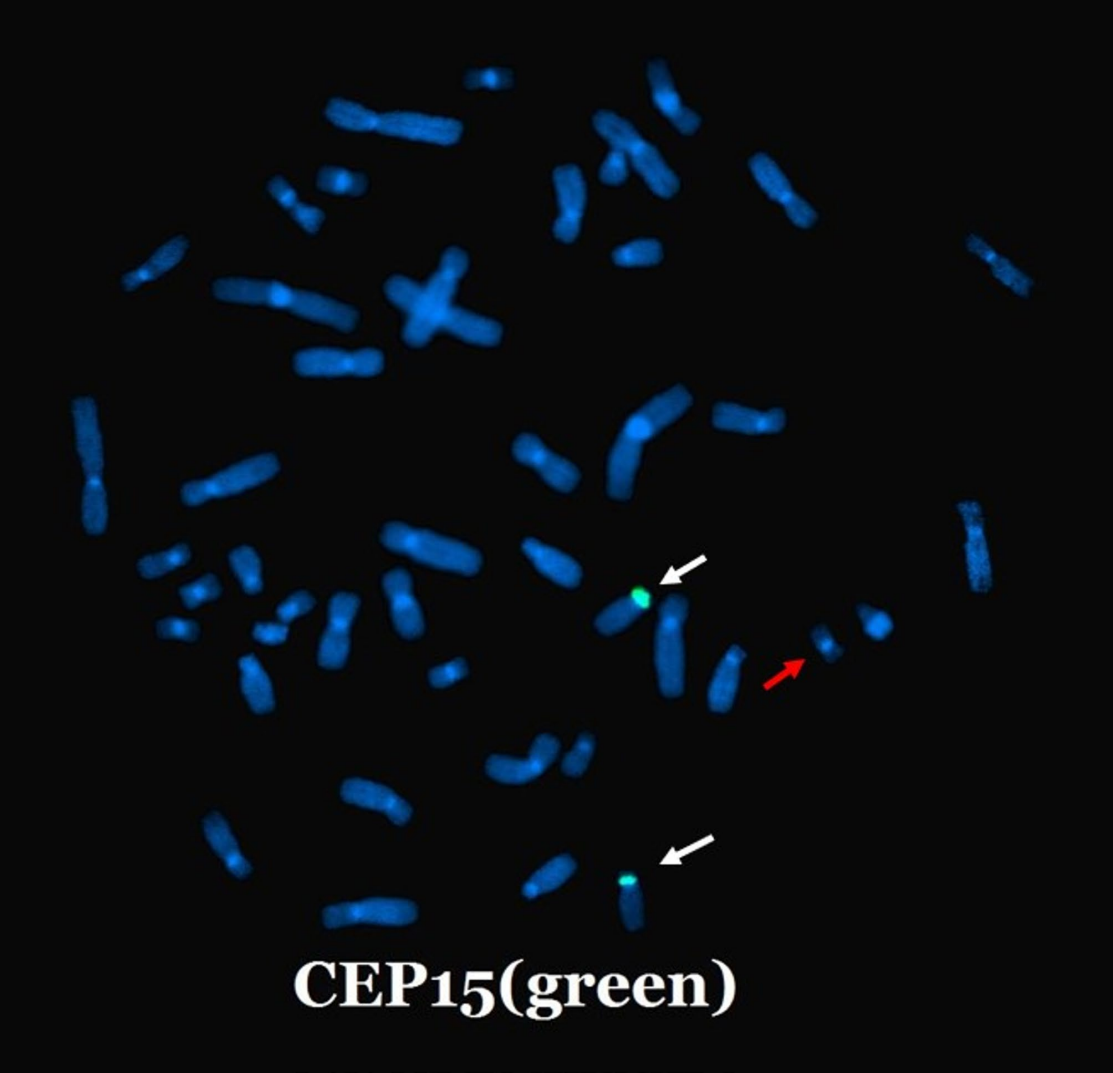
Fluorescence in situ hybridization performed on paternal peripheral blood utilizing the centromeric probe CEP15 pertinent to chromosome 15 shows that the identified marker chromosome (red arrow) does not originate from chromosome 15

To determine the origin of the marker chromosome, FISH analysis with the D15Z1 probe revealed significant polymorphism in the centromeric region of the chromosome 15 and confirmed that the marker chromosome was not derived from chromosome 15 (Fig. 5).

**Fig. 6:**
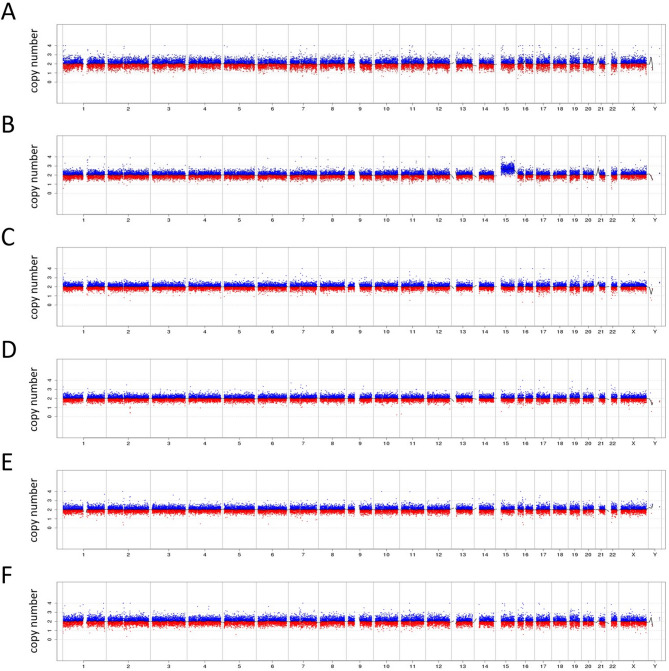
Examination of six placental biopsy samples (**A-F**) via CNV-seq identifies trisomy 15 in a single specimen (B), while the rest are normal. These findings indicate a mosaicism for trisomy 15 in the placenta

Given these results, the family received genetic counseling and chose to terminate the pregnancy. Following the termination, six placental samples uniformly distributed were collected for CNV sequencing (CNV-seq), confirming the initial suspicion of trisomy 15 in NIPT (Fig. 6). The procedure of CNV-seq was as follows. DNA was extracted from the placenta and hydrolyzed into fragments. The DNA library was constructed and was subjected to high-throughput 36 bp single-end sequencing on Illumina NextSeq 500 platform. All reads were aligned and analyzed using the parallel alignment software (Berry Genomics). Using a unique algorithm, the normalized copy number was shown on the y-axis. Therefore, the CNV of each chromosome could be identified through the graph (Fig. 6).

## Discussion

Advanced maternal age is associated with a higher incidence of chromosomal nondisjunction, which is a critical factor in this case study [15]. Our findings indicate that maternal age may increase the risk of chromosomal abnormalities and introduce complex compensatory mechanisms like trisomy rescue, potentially leading to discrepancies between non-invasive prenatal testing (NIPT) and confirmatory diagnostic tests [16].

**Fig. 7:**
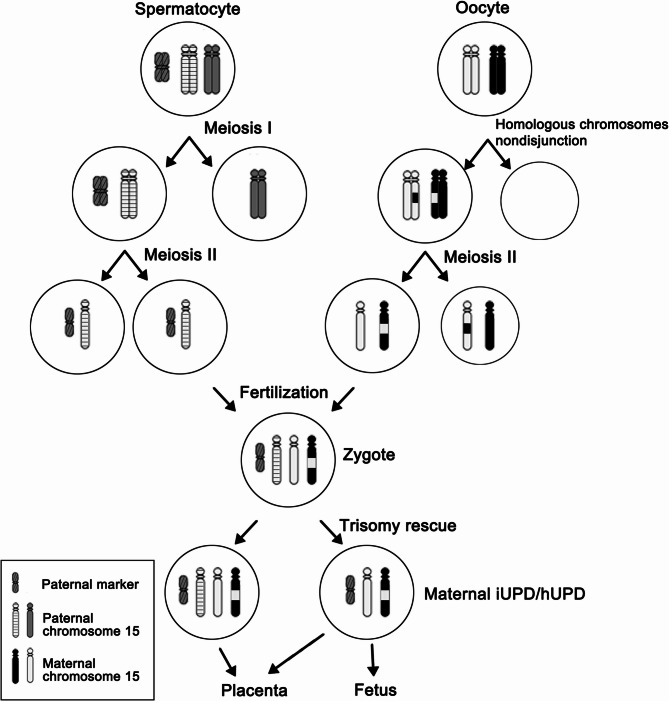
The possible mechanism of the formation of the maternal UPD with the paternal marker in the fetus

The analysis suggests that the fetus underwent an incomplete trisomy rescue, which is a postzygotic cellular correction process, resulting in mosaicism of both trisomic and disomic cell populations. This mosaicism could account for the fetal development and explain the initial high-risk NIPT result followed by the subsequent normal karyotype and CMA findings. The possible mechanism of the formation of the maternal UPD with the paternal marker of the fetus is illustrated in Fig. 7. The meiosis I nondisjunction created two homologous chromosome 15. Subsequent meiotic recombination occurred between the two homologous chromosomes. After meiosis II, the ovum contained two chromosome 15 with segmental heterodisomy and isodisomy. The postzygotic trisomy rescue leaded to the loss of paternal chromosome 15. As a result, the fetus presented maternal iUPD and hUPD of chromosome 15. The 15q11.2-q13 critical region remains isodisomic in our case, maintaining the complete maternal methylation pattern essential for PWS pathogenesis.

Clinicians ought to carefully interpret high-risk trisomy findings from NIPT as NIPT could indicate very complex fetal conditions, such as mosaicism, the emergence of marker chromosomes, or UPD, especially in the presence of incongruent chromosomal findings [17].

The identification of loss of heterozygosity (LOH) via CMA needs thorough investigation to rule out UPD. Failing to meticulously analyze high-risk NIPT results or identify LOH may conceal a UPD condition, which leads to severe clinical consequences, especially for chromosomes involved in imprinting disorders. Chromosomes 6, 7, 11, 14, 15, and 20 are of particular importance, requiring thorough investigation to exclude the possibility of UPD in prenatal diagnosis.

Our case identified a marker chromosome devoid of autosomal material, which was paternally inherited and not associated with chromosome 15. Previously reported cases involved marker chromosomes originating from the X chromosome and chromosome 22, respectively [18, 19]. While marker chromosomes are frequently associated with chromosome 15, our CMA revealed no duplications of euchromatic segments, indicating a possible derivation from another acrocentric chromosome within the D/G group (i.e., chromosome 13, 14, 15, 21, and 22). However, the precise characterization of the marker was hindered by the lack of available specific probes.

Small supernumerary marker chromosomes (sSMCs) most commonly originate from acrocentric chromosomes (especially 15), with mechanisms including U-type exchanges or incomplete trisomy rescue [20]. In prenatal diagnosis, sSMCs were observed in 0.075% of consecutively collected cases [21]. Even without detectable euchromatin, sSMCs may indicate the disruption of imprinting control regions (ICRs), particularly relevant for chromosome 15 [22].

The observed discordance between NIPT results and fetal diagnostic findings can be attributed to the biological characteristics of NIPT. Since NIPT analyzes cell-free DNA primarily derived from placental trophoblasts, the detection of a high-risk trisomy 15 result reflects the presence of trisomic cells in the placenta that failed to undergo trisomy rescue. In contrast, fetal cells in the amniotic fluid, having completed trisomy rescue, demonstrate a normal karyotype and CMA result. This phenomenon represents a classic case of confined placental mosaicism (CPM). From a clinical perspective, these findings underscore the importance of considering the possibility of CPM when interpreting discordant results between NIPT and invasive diagnostic testing. Such discrepancies highlight the need for comprehensive genetic counseling and confirmatory diagnostic procedures in cases of positive NIPT screening.

In conclusion, this case offers valuable perspectives, and highlights the critical need for molecular diagnostics in prenatal healthcare.

## Data Availability

No datasets were generated or analysed during the current study.
